# The behaviour in rats of tumour sublines of low and high malignancy.

**DOI:** 10.1038/bjc.1968.45

**Published:** 1968-06

**Authors:** A. E. Williams, R. S. Lowery, H. Smith

## Abstract

**Images:**


### THE BEHAVIOUR IN RATS OF TUMOUR SUBLINES OF LOW AND HIGH MALIGNANCY

A. E. WILLIAMS, R. S. LOWERY* AND H. SMITH

*From the Department of Microbiology, The University of Birmingham, Birmingham,* 15

Received for publication April 1, 1968

In continuation of studies of malignancy by methods comparable to those used to investigate microbial virulence, this paper describes comparative studies of the intraperitoneal growth and blood and visceral invasion of the two sublines of differing malignancy derived from a rat ascites tumour (Smith, Williams, Lowery and Keppie, 1968). The sublines were obtained by tumour progression, during passage of a 3,4-benzopyrene induced tumour: the 7th passage cells were the subline of low malignancy (WBP1(A)) and those of the 21st passage the subline of high malignancy (WBP1(V)).

Although studies of intraperitoneal growth (Klein and Révész, 1953; Andreev *et al.*, 1966; Lala and Patt, 1966) and blood and visceral invasion (Kinsey, 1960; Wheatley and Ambrose, 1964; Fisher and Fisher, 1965; Weiss, 1967) of tumours have been described, the authors are unaware of any on closely related sublines comparable to those described here. Furthermore, little attention has been paid to the early stages of tumour growth following intraperitoneal inoculation. These stages have been investigated here, as differences in behaviour between the sublines might indicate different capacities for invasion and combating possible host defence mechanisms, comparable to those recognized between virulent and avirulent strains of pathogenic bacteria (Burrows and Bacon, 1954; Stanier, Doudoroff and Adelberg, 1963). In addition, the amount of each subline present in animals at death has been assessed, thereby indicating the relative " toxicity " of the sublines (cf. similar consideration in the bacterial field—Smith, 1958).

### MATERIALS AND METHODS

### *Rats*

The inbred black and white hooded strain (Chester Beatty Research Institute) was bred under specific pathogen-free conditions. At intervals, the isogenicity of the animals was checked by skin grafting.

### *Tumour sublines WBP1(A) and WBP1(V)*

These were prepared and stored as described previously (Smith *et al.*, 1968). The mean death times of rats receiving $4 \cdot 5 \times 10^7$ viable cells intraperitoneally was 31·1 days for WBP1 (A) and 15·3 days for WBP1(V). The constancy of the results of the determinations (Smith *et al.*, 1968), made at intervals concurrently with the present experiments, indicated the continuing compatibility of both sublines of the tumour with successive generations of rats.

* Present address: Department of Botany and Zoology, Sir John Cass College, Jewry Street, London, E.C.3.

*Inoculation of tumour sublines*

In comparative studies, standard doses ($1 \times 10^7$ viable cells in 1·0 ml. Tyrode's-gelatin-citrate solution (TGC, see Smith *et al.*, 1968) of the two sublines were inoculated intraperitoneally into randomized groups of rats (250–300 g.). After all animals had been injected the viability of the remainder of the inoculum was $>95\%$. A third group received only TGC solution (1·0 ml.).

*Estimation of the number of free tumour and host cells in the peritoneal cavity and blood at intervals after injection of the tumour sublines*

At intervals, animals were killed by ether vapour and the numbers of tumour and host cells in the peritoneal contents and blood were estimated by a combination of total and differential counts.

*Peritoneal cavity.*—The total populations of free host and tumour cells were determined by the method of Révész and Klein (1954) which was adapted for work in rats.

The cells were centrifuged from the suspensions used for the total counts, suspended in calf serum (1 drop) and smeared. The smears were fixed (ether/ethanol 1 : 1 by vol.; 10 min.) and stained by the Papanicolaou method, excluding the orange G counterstain. Using an eyepiece net graticule and a $\times 400$ magnification, the number of tumour and host cells (excluding contaminating erythrocytes) was counted in a strip, one graticule width across, through the centre of each smear (1000–3000 cells). The uneven distribution of tumour cells (more occurring at the middle than at the edge) necessitated counting completely across a smear. The identities of the slides were concealed from the person counting. Cytological criteria distinguishing tumour cells (Fig. 1) from host cells were a large nuclear/cytoplasmic ratio, an eccentrically placed nucleus, 1–3 prominent nucleoli and a characteristic staining reaction: the cytoplasm of tumour cells was grey in comparison with the green cytoplasm of host cells. There were no morphological differences between sublines WBP1(A) and WBP1(V). Counts of tumour cells in the presence of host cells could be made with acceptable accuracy throughout tumour growth in the peritoneal cavity, even initially when the proportion of host cells was high. Thus, triplicate differential counts on slides from a typical experiment (Table I) show the ratio greatest tumour percentage/least tumour percentage as $> 1.5$ for only 5 % of the slides. In two further experiments this ratio was $> 1.5$ for 5 % and 19 % of the slides. This accuracy was acceptable and hence tedious replicate counts were not done routinely. Gross errors were, of course, accounted for in the variation of the mean tumour count of the whole group of animals.

*Blood.*—Blood was taken from the end of the tail or from the heart and erythrocyte and white cell (including tumour cells) counts made in a haemocytometer. Blood smears were fixed in methanol and stained with Giemsa stain (Hopkins and Williams " Revector " Giemsa stain (1 vol.), M/15 phosphate buffer pH 7·2

---

EXPLANATION OF PLATE

FIG. 1.—Intraperitoneal smear of rat with WBP1 ascites tumour. Papanicolaou stain. $\times 1000$.

(a) Five tumour cells, showing large nuclear-cytoplasmic ratio and multiple nucleoli.
(b) Three tumour cells (arrowed) and three host cells.

1

Williams, Lowery and Smith.

TABLE I.—*Triplicate Differential Counts for Tumour Cells on Samples of Smears of the Intraperitoneal Contents of Rats following the Inoculation of Sublines WBP1(A) and WBP1(V)*

| Hours after inoculation | WBP1(A) % Tumour in Count 1 | 2 | 3 | Highest %/Lowest % | WBP1(V) % Tumour in Count 1 | 2 | 3 | Highest %/Lowest % |
|---|---|---|---|---|---|---|---|---|
| 0·5 | — | — | — | — | 37·8 | 45·2 | 37·5 | 1·2 |
| 8 | 7·0 | 9·1 | 10·5 | 1·1 | 11·9 | 12·0 | 10·3 | 1·2 |
| 16 | 15·1 | 13·5 | 15·9 | 1·2 | — | — | — | — |
| 24 | 6·4 | 7·5 | 7·4 | 1·2 | 9·4 | 11·6 | 13·5 | 1·4 |
| 29 | 11·9 | 12·7 | 12·9 | 1·1 | — | — | — | — |
| 32 | — | — | — | — | { 1·4 | 1·8 | 1·6 | 1·3 |
| | | | | | 4·2 | 5·4 | 6·1 | 1·5 |
| 48 | — | — | — | — | { 15·4 | 20·5 | 18·8 | 1·3 |
| | | | | | 3·2 | 4·8 | 7·1 | 2·2 |
| 52 | 21·2 | 34·8 | 34·1 | 1·6 | — | — | — | — |
| 70 | 40·0 | 48·2 | 47·1 | 1·2 | 45·4 | 62·1 | 65·4 | 1·4 |
| 99 | 81·0 | 83·2 | 71·4 | 1·2 | 63·8 | 74·0 | 70·9 | 1·2 |
| 124 | 87·8 | 78·8 | 74·4 | 1·2 | — | — | — | — |
| 190 | — | — | — | — | 81·8 | 85·9 | 81·4 | 1·1 |
| 243 | — | — | — | — | 78·9 | 85·4 | 86·0 | 1·1 |
| 315 | 96·1 | 92·3 | 93·1 | 1·0 | — | — | — | — |

(9 vol.); 40 min.). Differential counts of tumour and host white blood cells were made as described for the peritoneal contents: a magnification of ×1000 was used and the morphological features of the cancer cells were essentially those described for the Papanicolaou stained smears, except for the differential staining of the cytoplasm. Again, tumour cells could be distinguished from host cells with acceptable accuracy. Triplicate counts on a proportion of the slides from each experiment gave the ratio greatest tumour percentage/least tumour percentage as > 1·5 in only 13·6 %, 0 % and 10 % of the slides.

*Weights of tissues invaded by the tumour sublines*

The spleen, mesenteric lymph node and thymus were invaded by the tumour. Also, a solid abdominal tumour, inseparable from the pancreas, grew as a grape-like mass below the stomach. As an indication of tumour growth in these areas, the wet weights of the tissues, as percentages of body weight, were obtained at intervals. The mean wet weight of the pancreas (0·68 g.) of control animals was deducted from the total solid tumour weight and the corrected tumour weight expressed as a percentage of body weight.

### RESULTS

*Comparison of the changes in free tumour cell populations of sublines WBP1(A) and WBP1(V) in the peritoneal cavity*

Groups of rats received WBP1(A) cells, WBP1(V) cells and TGC solution alone, and were sampled as described in Methods. Six rats receiving each tumour subline and 2 control animals were examined every 8 hours for the first 48 hours when the intraperitoneal tumour cell concentration was low. Subsequently 2 experimental rats (for each subline) were examined every day and one control rat every other day.

The data from three similar experiments reveal the changes in total number of free tumour cells with time for both sublines of WBP1 (Fig. 2 and 3). The viability of peritoneal cells (host and tumour) was always >95 %. The data from each of the 3 experiments was examined using an Analysis of Variance procedure on the logarithms of the tumour cell numbers. In all experiments, there was a significant difference ($p < 0{\cdot}05$) between the shapes of the curves described by the data from the WBP1(V) subline and by that from the WBP1(A) subline. Following injection a rapid decrease occurred in the number of free tumour cells in the peritoneal cavity. The decrease was more precipitous and reached a lower level ($1{\cdot}5 \times 10^6$ cells) with WBP1(V) cells than with WBP1(A) cells (minimum $3 \times 10^6$ cells); within 30 minutes of injection there was a twofold decrease in the number of free WBP1(V) cells, whereas WBP1(A) cells had not decreased from the dose injected. After *ca.* 24 hours the free tumour cell populations of both sublines reached a minimum and then increased rapidly over the following 4 days,

Fig. 2.—The changes in free intraperitoneal tumour cell populations of tumour sublines WBP1 (A), (△), and WBP1(V), (○). The data is derived from three similar experiments. Each point represents the mean of data from one experiment (6 rats per point from 0–2 days, and 2 rats per point thereafter). The curves drawn to the points have been fitted by eye. In each of the three experiments an Analysis of Variance of the logarithms of the tumour cell numbers showed a significant difference ($p < 0{\cdot}05$) between the shapes of the curves described by the data for the two tumour sublines.

the WBP1(V) cells more so than the WBP1(A) cells. The increases in tumour populations were not exponential; the doubling times increased as growth proceeded. This slowing down was more apparent with WBP1(A) subline than with WBP1(V) subline and therefore, although both sublines attained the same maximum population of *ca.* 2 × $10^9$ cells, the WBP1(V) cells reached this concentration after 10 days whereas WBP1(A) cells took 13 days. A gradual decrease in the peritoneal population then followed until the deaths of the rats carrying WBP1(V) cells at *ca.* 16 days and of those carrying WBP1(A) cells at *ca.* 30 days.

*Invasion of the blood*

The tumour cell concentrations in the blood following intraperitoneal injection with WBP1(V) or WBP1(A) cells were measured throughout tumour growth in 3

FIG. 3.—The changes in free intraperitoneal tumour cell populations of tumour sublines WBP1 (A), (△), and WBP1(V), (○), during the first 6 days after injection. The data are identical to those shown in Fig. 1, but have been plotted using an expanded time scale to show the detail of the initial decrease in free tumour cell numbers. Each point represents the mean (0–2 days: 6 rats per mean; 2–6 days: 2 rats per mean) of data from one experiment. The curves drawn to the points have been fitted by eye. In each of the three experiments an Analysis of Variance of the logarithms of the tumour cell numbers showed a significant difference ($p < 0{\cdot}05$) between the shapes of the curves described by the data for the two tumour sublines.

separate experiments. Tumour cells were detectable in the blood from 5–6 days after intraperitoneal injection. The concentration increased rapidly, that of rats carrying WBP1(V) cells reaching a given value *ca.* 1 day before that of rats carrying WBP1(A) cells. The maximum tumour cell concentration in the blood (*ca.* $1{\cdot}0 \times 10^8$ cells/ml.) was attained just before death (14 days) in rats with WBP1(V) subline and at 16–17 days in rats with WBP1(A) subline. The concentration in those rats surviving with WBP1(A) subline then decreased to *ca.* $2 \times 10^7$ cells/ml. until their deaths (at *ca.* 30 days).

The erythrocyte concentration remained at the control level ($7\text{–}8 \times 10^9$ cells/ml.) for 8 days after inoculation of either subline (Fig. 4). That of rats with WBP1(V) subline then rapidly decreased until the rats died when there were $3\text{–}4 \times 10^9$ RBC's/ml. In rats with WBP1(A) subline, the RBC concentration also decreased between 11 and 18 days post inoculation to *ca.* $5 \times 10^9$ RBC's/

FIG. 4.—The changes in erythrocyte concentration in the blood of rats injected with tumour sublines WBP1(A), (△; fiducial limits (95%)⟙) and WBP1(V), (○; fiducial limits (95%) =). Each point represents the mean of pooled data obtained from three similar experiments. Fiducial limits are shown for points after day 10 when significant decreases in RBC concentrations from the mean control value were apparent.

ml.; subsequently, a partial but significant recovery to 6 $\times 10^9$ RBC's/ml. was maintained until just before death. An increase in the proportion of reticulocytes and large erythrocytes was noted with both tumour sublines.

*Changes in host cell populations in the peritoneal cavity and blood of rats after injection of sublines WBP1(A) and WBP1(V)*

Following injection of tumour cells a five- to tenfold increase in the normal leucocyte populations of the peritoneal cavity and blood was noted parallelling the increase in tumour cell numbers. There were no significant differences between the total host responses to the 2 sublines in the peritoneal cavity or in the blood.

*Lymphatic and visceral invasion*

The weights of the spleen, mesentric lymph node and thymus increased relative to body weight after 5–6 days post inoculation continuing until death of the animal (Table II). There were no significant differences between the weights of organs from rats with subline WBP1(V) and WBP1(A) up to the death of WBP1(V) inoculated animals (*ca.* 15 days). Thereafter, the weights of organs in rats with WBP1(A) cells continued to increase and became significantly greater in relation to body weight than were the same organs at death in rats with WBP1(V) cells.

TABLE II.—*The Increases in Relative Weights of Organs and Solid Abdominal Tumour during the Growth of Sublines WBP1(V) and WBP1(A)*

| Tissue | Time post inoculation days | $\frac{\text{g. tissue}}{\text{g. body weight}} \times 100$ WBP1(V) | WBP1(A) |
|---|---|---|---|
| Spleen | 0 | 0·18±0·03* | 0·18±0·01 |
| | 15 | 0·61±0·08 | 0·72±0·05 |
| | 17 | — | 0·90±0·06 |
| | 30 | — | 0·66±0·21 |
| Thymus | 0 | 0·08±0·04 | 0·08±0·02 |
| | 15 | 0·87±0·36 | 0·83±0·18 |
| | 20 | — | 1·42±0·41 |
| | 30 | — | 1·39±0·62 |
| Mesenteric lymph node | 0 | 0·19±0·10 | 0·23±0·08 |
| | 15 | 0·41±0·20 | 0·33±0·21 |
| | 25 | — | 1·74±0·62 |
| | 30 | — | 1·87±0·73 |
| Solid abdominal tumour | 0 | 0 | 0 |
| | 15 | 3·54±0·53 | 5·23±0·78 |
| | 20 | — | 8·49±0·85 |
| | 30 | — | 6·82±2·58 |

* Fiducial limits (95%).

In animals inoculated with either tumour, the solid abdominal tumour attached to the pancreas became apparent after 5–6 days. The weight of this, relative to body weight, was significantly greater in rats with WBP1(A) subline at *ca.* 15 days, when the rats with WBP1(V) subline were dying and continued to increase thereafter (Table II).

### DISCUSSION

The changes in the free ascites tumour cell population described here were similar to those observed with other ascites tumours (Klein and Révész, 1953; Andreev *et al.*, 1966; Lala and Patt, 1966; Maruyama and Knuth, 1966). After an initial decrease, modified exponential growth occurred similar to that described by Laird (1964). However, although the growth curves for the 2 sublines were of similar pattern they differed in detail. The initial decrease in free tumour cells occurred more rapidly with WBP1(V) cells than with WBP1(A). Three factors, which may contribute to these initial decreases are discussed below.

Firstly, more WBP1(V) cells than WBP1(A) cells may have died due to a differential action of non-specific host defence mechanisms (phagocytosis and extracellular lysis). This seemed unlikely, because the proportion of damaged cells did not increase over that of the inoculum and no large scale phagocytosis of cellular debris was observed. Secondly, tumour cells could have attached to the peritoneal lining and to the abdominal viscera. Such an attachment may be a prelude to invasion (Wheatley and Ambrose, 1964; Wheatley and Easty, 1964). Different surface properties (Abercrombie and Ambrose, 1962) of the two tumour sublines might have affected attachment to normal tissue surfaces, leading to the observed differential disappearance. Thirdly, WBP1(V) cells may have drained to the lymphatics more rapidly than WBP1(A) cells. Although early direct counts of tumour cells in lymph or blood were not practicable, a more rapid invasion of WBP1(V) might be detected by inoculation of peripheral blood into fresh animals (*cf.* Greene, 1965; Baker and Wood, 1967; Rosso, Donelli and Garattini, 1967).

Several factors may have affected the rates of increase of the 2 free tumour cell populations during the subsequent phase of growth. Decreases in the mitotic rates and in the growth fractions may have contributed to the retardation of population increase (Lala and Patt, 1966; Mendelsohn, 1962; Baserga, 1963). The lack of an essential nutrient, which can explain the rapid cessation of exponential growth in bacterial cultures, is unlikely to be the cause of such decreases (Laird, 1964). More likely, growth inhibitors may accumulate, as the result of host tissue damage or as a product of the tumour cells. A greater effectiveness of host defence mechanisms against the WBP1(A) cells could also cause the more rapid cessation of intraperitoneal growth observed with this subline.

Tumour cell population changes in the blood were similar to those in the peritoneal population. The survival of rats carrying WBP1(A) cells for a considerable period, during which the free ascites cell populations and tumour cell populations in the blood attained and declined from maximum values, supports the argument of Goldie, Watkins, Powell and Hahn (1952) that the ascites and blood tumour populations *per se* play little direct part in the pathogenesis of the disease and merely act as a reservoir of tumour cells.

The erythrocytic anaemia which developed in rats with WBP1(V) subline after 8 days and in rats with WBP1(A) subline after 11 days was striking. The terminal anaemia (*ca.* 50 %) in the rats with WBP1(V) subline may have contributed directly to the deaths of the animals. In the rats with WBP1(A), anaemia (*ca.* 35 %) was not as severe at the time when rats with WBP1(V) died and later decreased. Whether this improvement in the rats with WBP1(A) subline, and the decreasing tumour counts in the peritoneal cavity and blood, was an effect of the host response has not been resolved. If it was, it was of no avail because of the uninterrupted increase in the cachectic condition of the rats which terminated in death.

The extent of visceral invasion by these tumours may have been indicated by the increases in relative weights of the spleen, mesenteric lymph node, thymus and solid abdominal tumour. The spleen, mesenteric lymph node and thymus increases could be due partly to an increase in the lymphoid elements in response to tumour growth. However, histological examinations revealed extensive tumour in these organs. At death there appeared to be significantly more of WBP1(A) subline present in the rats than WBP1(V) subline. In addition, there was more WBP1(A) present in *ca.* 15 days when the rats with WBP1(V) subline were dying. These findings suggested that subline WBP1(V) might be more "toxic" to the host than subline WBP1(A). The cause of the greater toxicity—earlier invasion of some vital site, more efficient debilitation of the host by utilization of nutrients or the production of toxic materials by the tumour (cf. Sylvén and Holmberg, 1965) is as yet unknown.

### SUMMARY

The intraperitoneal growth and blood and visceral invasion of more (WBP1(V)) and less (WBP1(A)) malignant sublines of a rat ascites tumour have been compared.

In the peritoneal cavity there was an initial decrease in free tumour cell numbers, more precipitous and reaching a lower level with WBP1(V) cells than with WBP1(A) cells. This was followed by a period of increase during which exponential growth of the population was increasingly retarded. The retardation of population growth rate was greater for WBP1(A) cells than for WBP1(V) cells. After the deaths of rats carrying WBP1(V) subline the free ascites tumour population decreased in rats carrying WBP1(A) subline.

A similar pattern of tumour cell increase in the blood was observed after 5 days. No differences were apparent between the tumour sublines up to the time of death of rats carrying WBP1(V) cells. Subsequently, a decrease in the concentration of circulating tumour cells occurred in the surviving rats carrying the WBP1(A) subline.

There was a large and progressive host cell response which did not appear to differ with the subline injected.

An increasing anaemia developed in all tumour bearing animals being more severe in rats carrying WBP1(V) cells than in those carrying WBP1(A) cells.

The weights of spleen, mesenteric lymph nodes, thymus glands and solid abdominal tumour indicated that more tumour had accumulated in rats dying from WBP1(A) cells (after approximately 30 days) than in animals dying from WBP1(V) cells (after approximately 15 days).

We wish to thank Miss S. M. Christie and Mr. M. S. Macbeth for technical assistance, and to acknowledge the advice given by Professor H. E. Daniels and Mr. R. Holder of the Department of Mathematical Statistics, University of Birmingham, and by Dr. J. Marchant. The project was supported by grants from the Central Organisation and Birmingham Council of the British Empire Cancer Campaign for Research.

### REFERENCES

Abercrombie, M. and Ambrose, E. J.—(1962) *Cancer Res.*, **22**, 525.

Andreev, V. M., Afanas'ev, G. G., Lipchina, B. P., Pelvina, I. I. and Emanuel, N. M.—(1966) *Dokl. (Proc.) Acad. Sci. U.S.S.R., Biol. Sci. Sect.*, **169**, 516.

Baker, R. R. and Wood, S.—(1967) *Surgery Gynec. Obstet.*, **124**, 742.

Baserga, A. R.—(1963) *Archs Path.*, **75**, 156.

Burrows, T. W. and Bacon, G. A.—(1954) *Br. J. exp. Path.*, **35**, 134.

Fisher, E. R. and Fisher, B.—(1965) *Acta cytol.*, **9**, 146.

Goldie, H., Watkins, F. B., Powell, C. and Hahn, P. F.—(1952) *Cancer Res.*, **12**, 92.

Greene, H. S. N.—(1965) *Acta cytol.*, **9**, 160.

Kinsey, D. L.—(1960) *Cancer, N.Y.*, **13**, 674.

Klein, G. and Révész, L.—(1953) *J. natn. Cancer Inst.*, **14**, 229.

Laird, A. K.—(1964) *Br. J. Cancer*, **18**, 490.

Lala, P. K. and Patt, H. M.—(1966) *Proc. natn. Acad. Sci. U.S.A.*, **56**, 1735.

Maruyama, Y. and Knuth, P.—(1966) *Growth*, **30**, 453.

Mendelsohn, M. L.—(1962) *J. natn. Cancer Inst.*, **28**, 1015.

Révész, L. and Klein, G.—(1954) *J. natn. Cancer Inst.*, **15**, 253.

Rosso, R., Donelli, M. G. and Garattini, S.—(1967) *Cancer Res.*, **27**, 1225.

Smith, H.—(1958) *A. Rev. Microbiol.*, **12**, 77.

Smith, H., Williams, A. E., Lowery, R. S. and Keppie, J.—(1968) *Br. J. Cancer*, **22**, 359.

Stanier, R. Y., Doudoroff, M. and Adelberg, E. A.—(1963) in " General Microbiology ", 2nd edition. London (Macmillan) p. 584.

Sylvén, B. and Holmberg, B.—(1965) *Eur. J. Cancer*, **1**, 199.

Weiss, L.—(1967) *in* ' The cell periphery, metastasis and other contact phenomena '. Amsterdam (North Holland Publishing Co.), pp. 289 and 315.

Wheatley, D. N. and Ambrose, E. J.—(1964) *Br. J. Cancer*, **18**, 730.

Wheatley, D. N. and Easty, G. C.—(1964) *Br. J. Cancer*, **18**, 743.


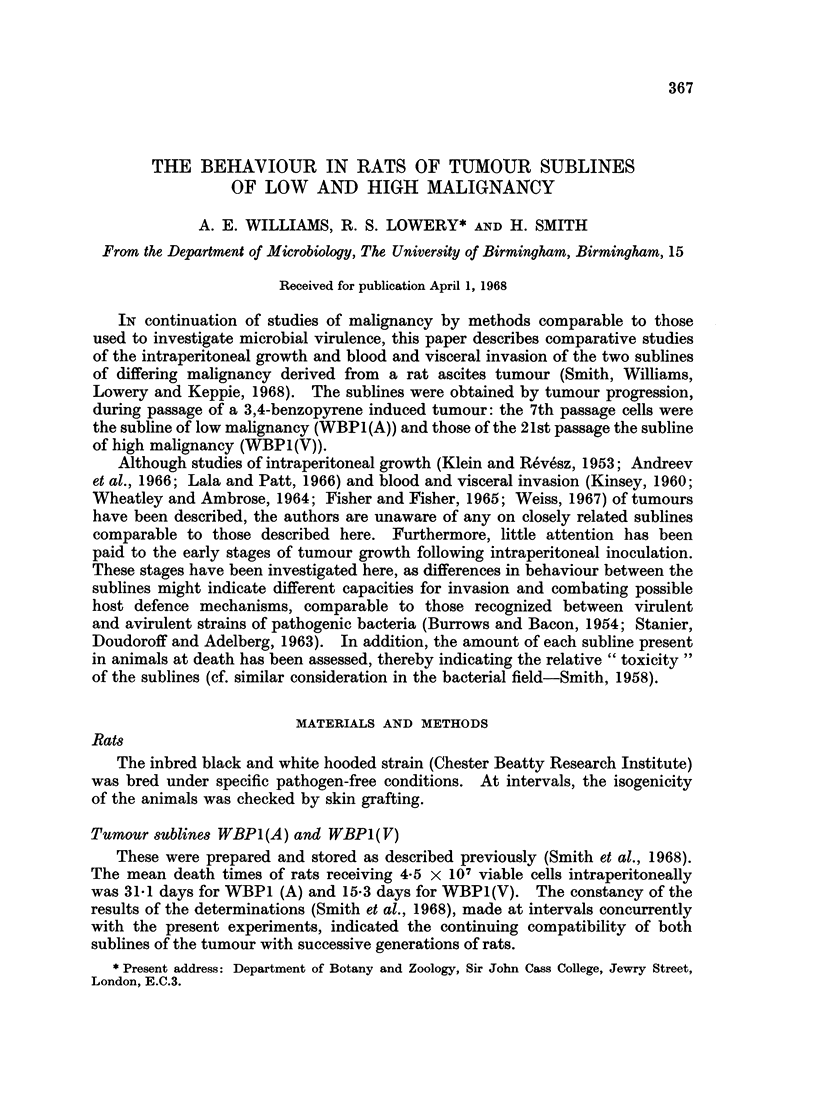

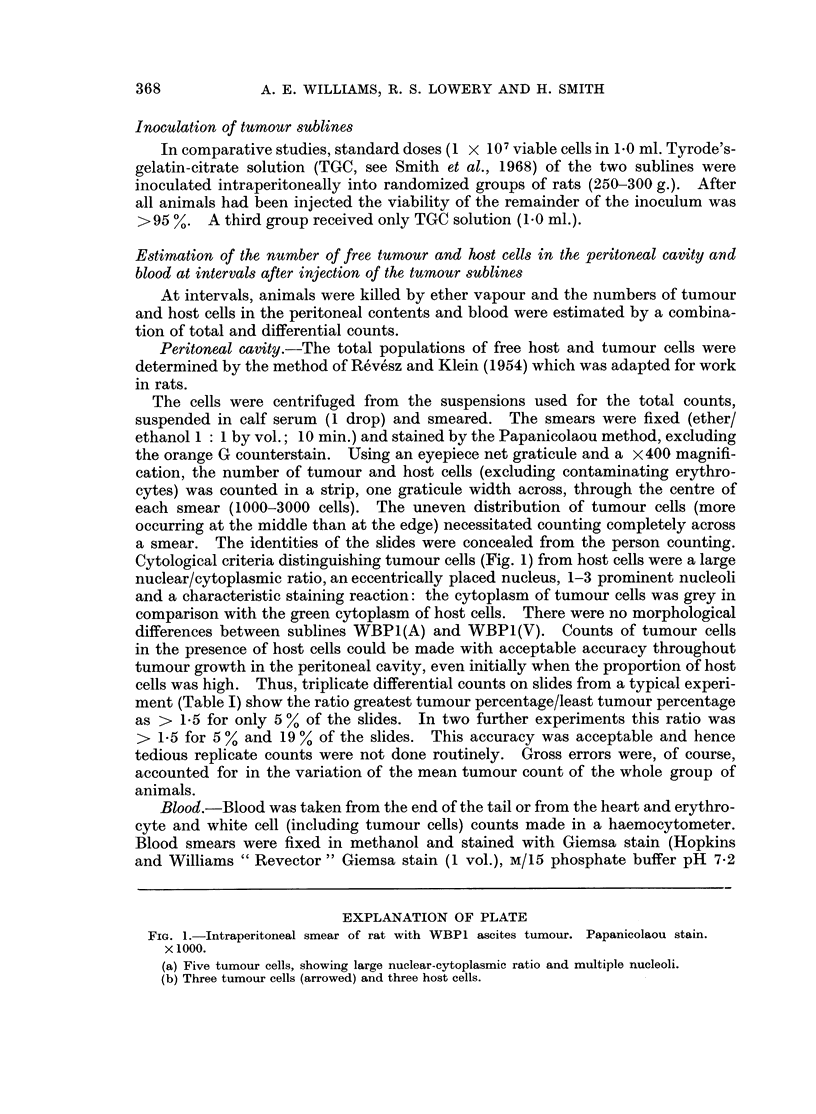

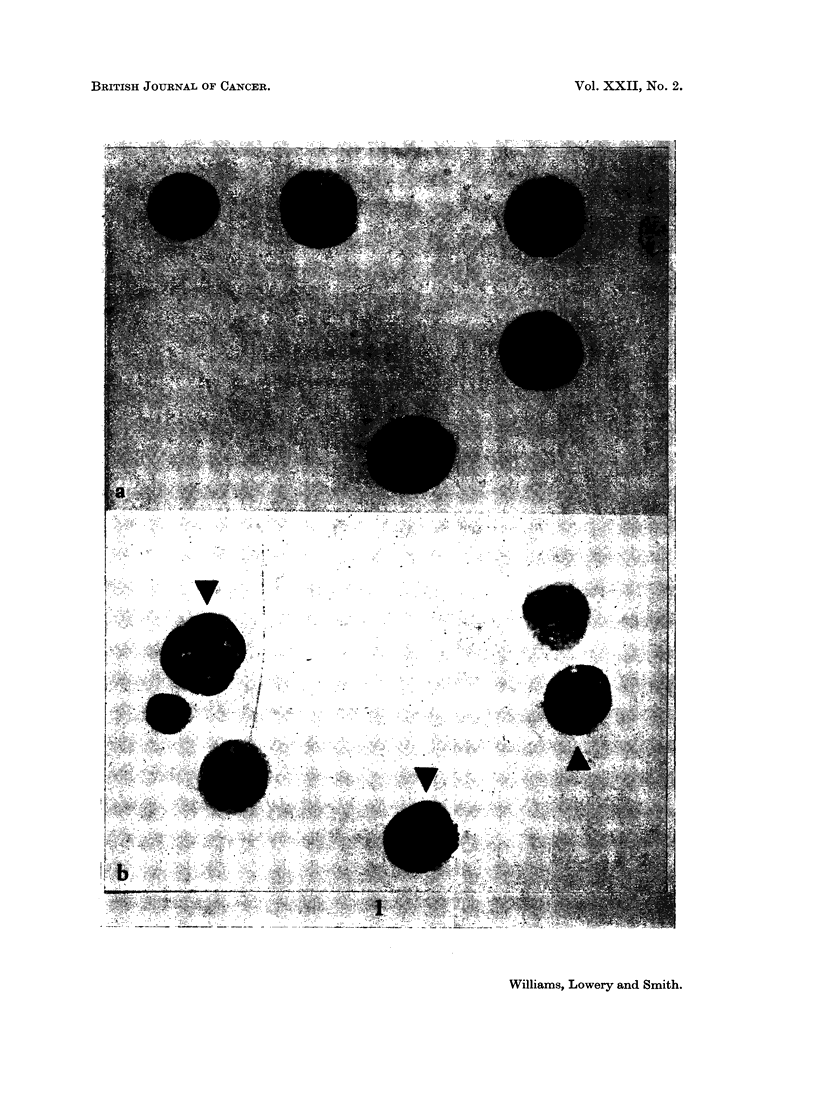

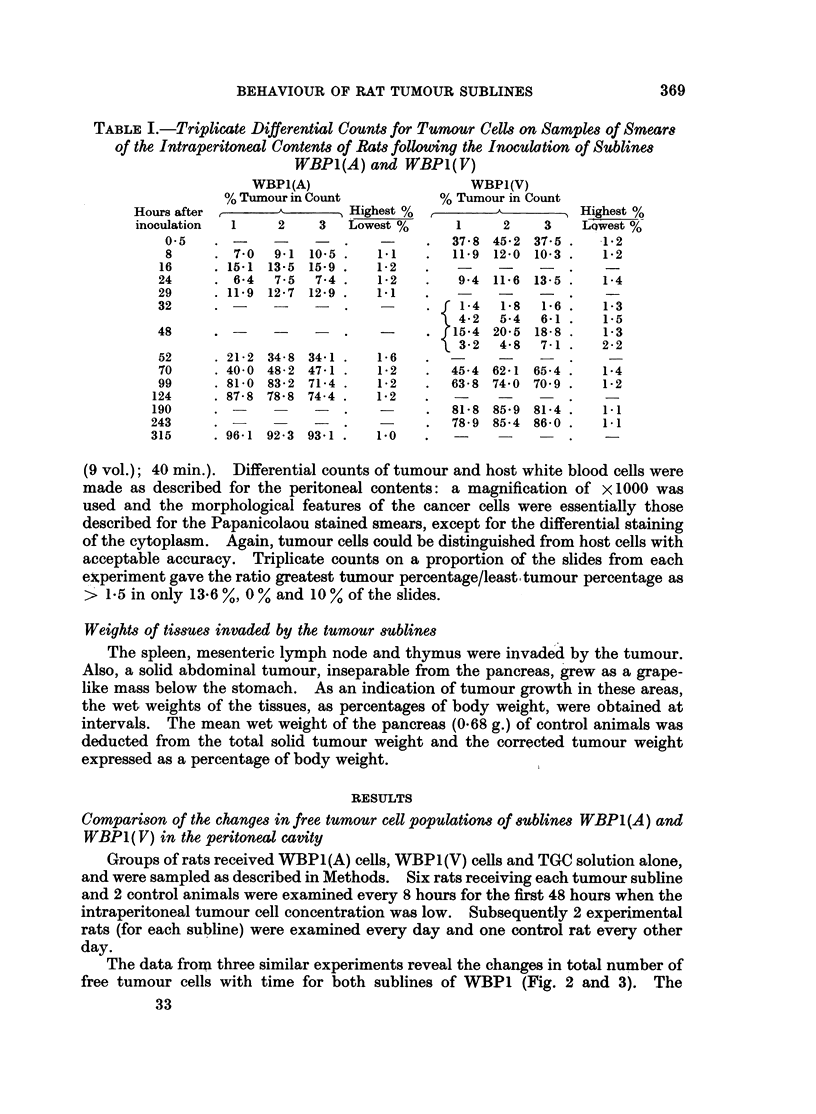

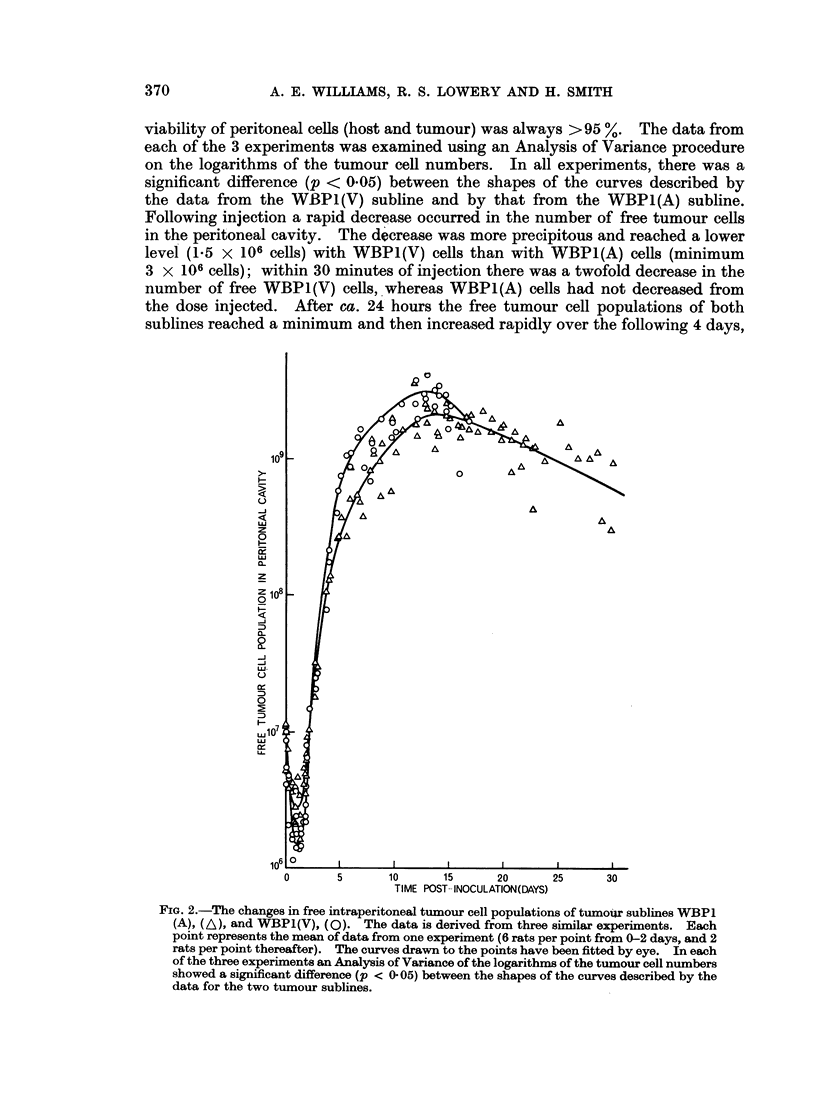

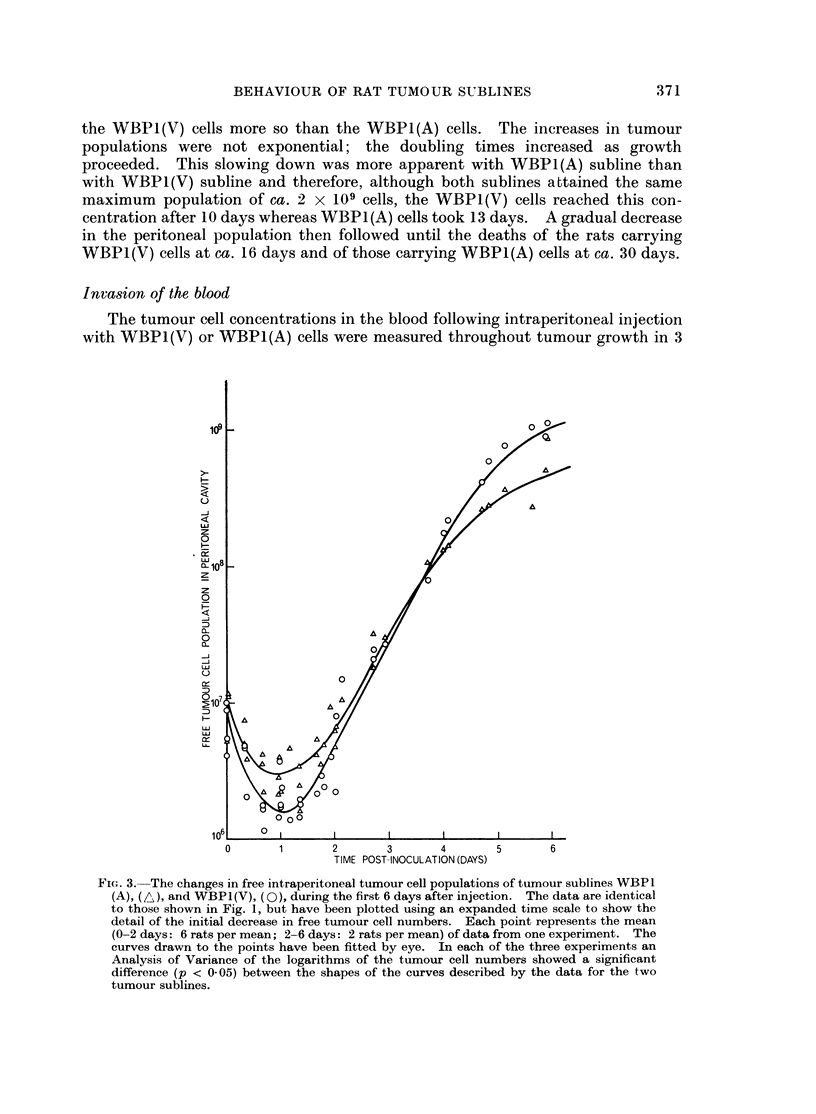

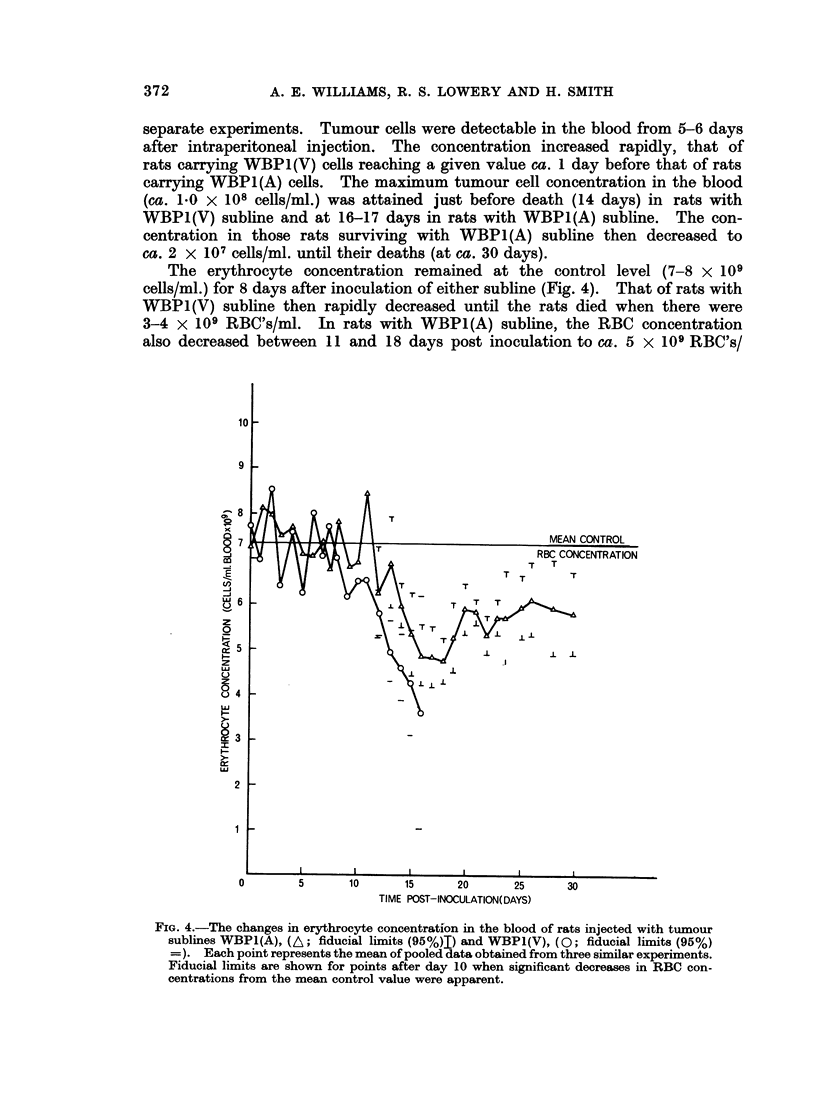

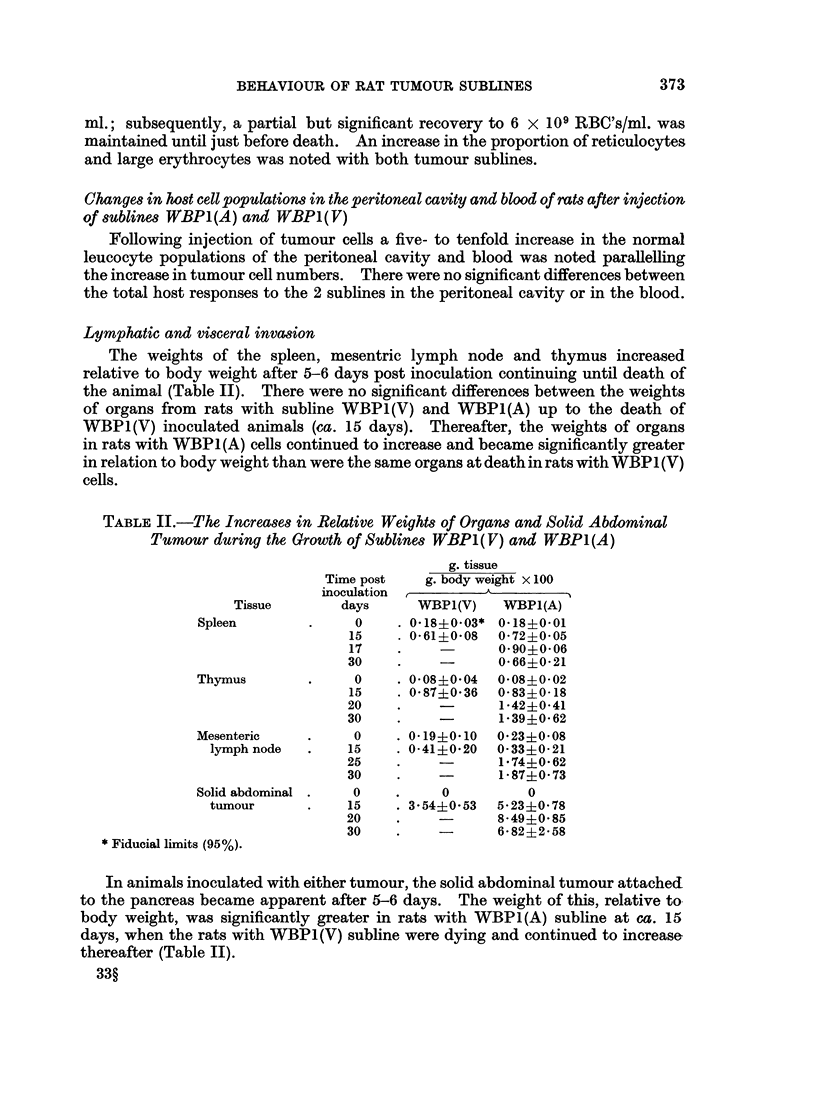

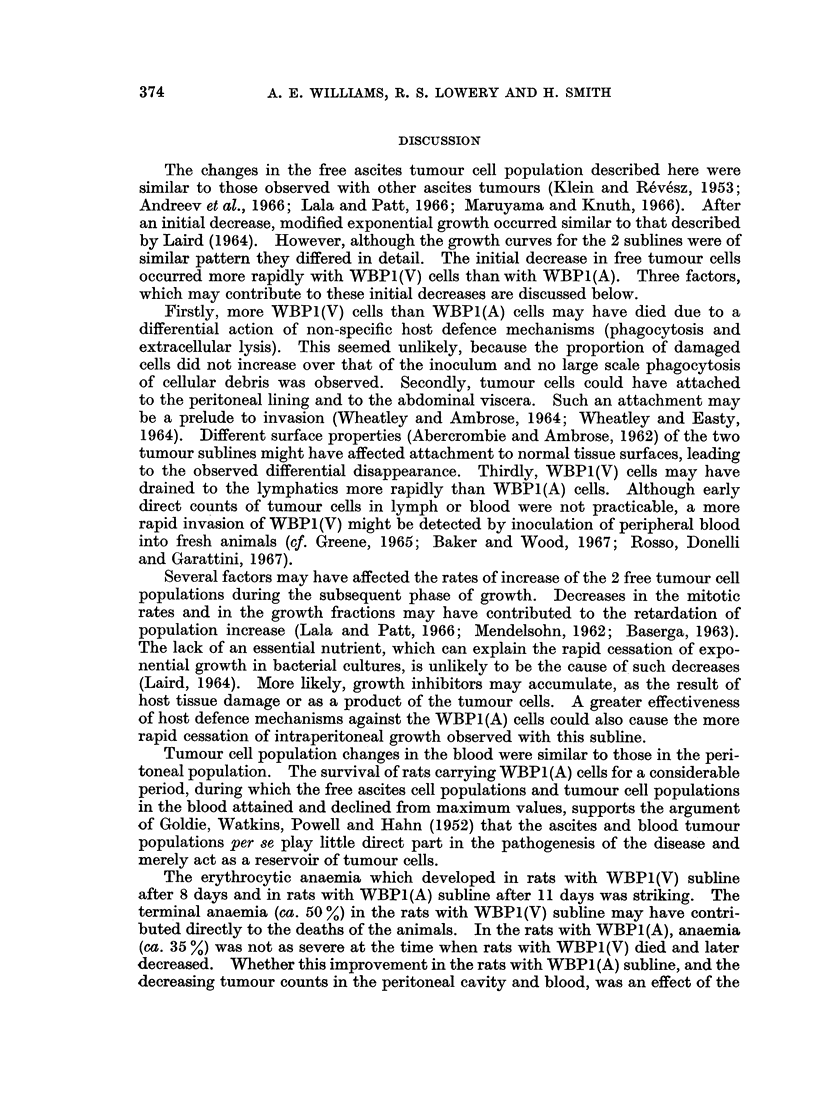

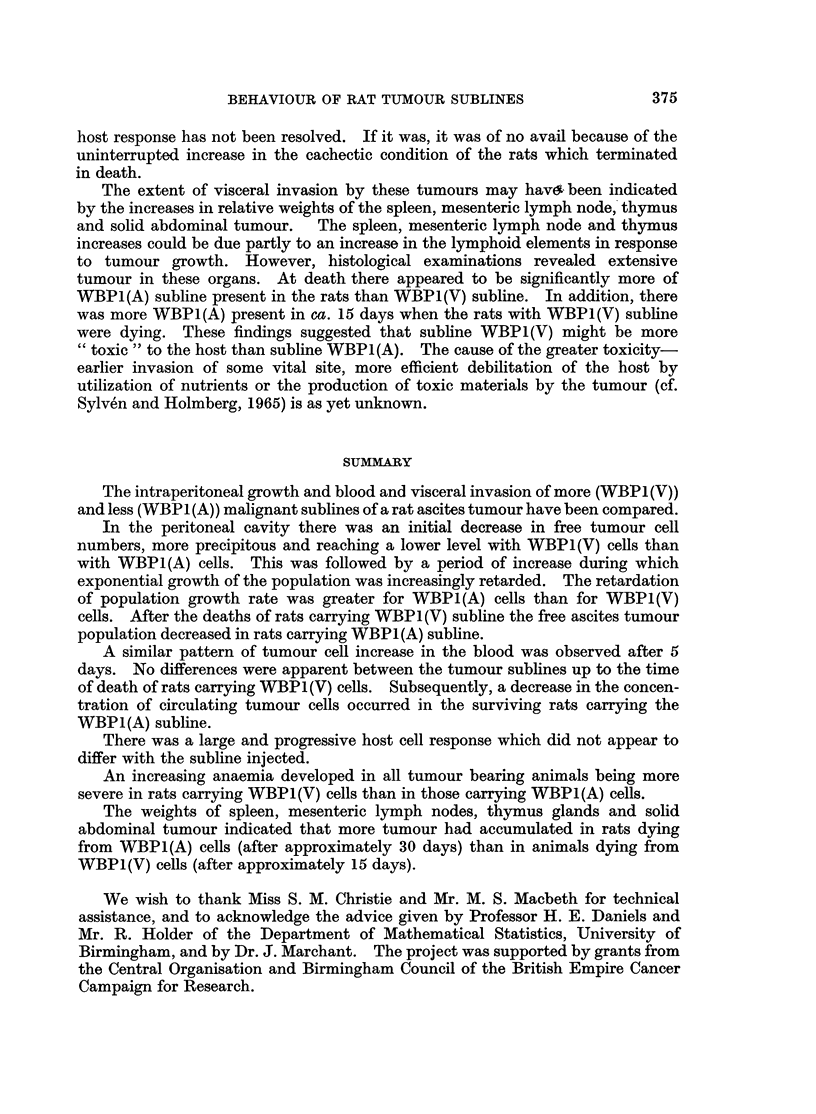

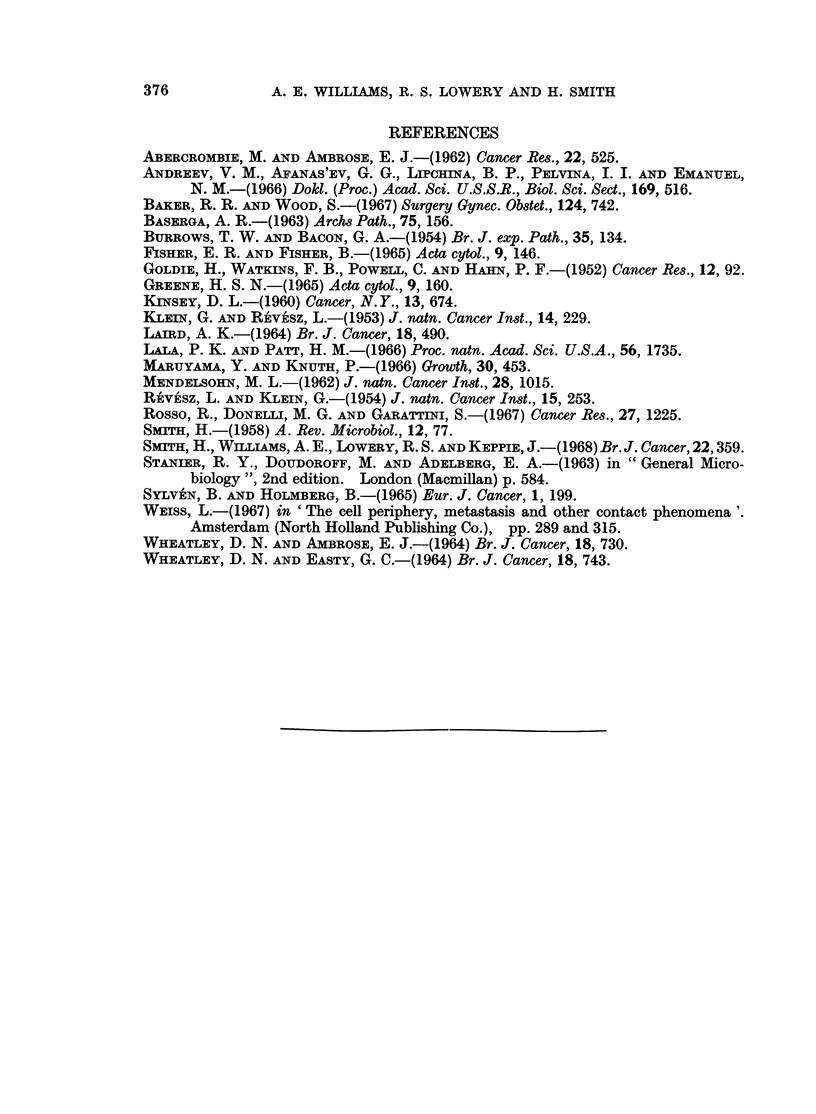


## References

[OCR_00558] ABERCROMBIE M., AMBROSE E. J. (1962). The surface properties of cancer cells: a review.. Cancer Res.

[OCR_00564] BASERGA R. (1963). Mitotic cycle of ascites tumor cells.. Arch Pathol.

[OCR_00566] BURROWS T. W., BACON G. A. (1954). The basis of virulence in Pasteurella pestis: comparative behaviour of virulent and avirulent strains in vivo.. Br J Exp Pathol.

[OCR_00569] FISHER E. R., FISHER B. (1965). EXPERIMENTAL STUDY OF FACTORS INFLUENCING DEVELOPMENT OF HEPATIC METASTASES FROM CIRCULATING TUMOR CELLS.. Acta Cytol.

[OCR_00572] GREENE H. S. (1965). A METHOD FOR DETERMINING THE PRESENCE OF TUMOR CELLS IN BLOOD AND ORGANS OF EXPERIMENTAL ANIMALS AND ITS APPLICATION TO THE PROBLEMS OF METASTASES AND RETENTION IN ORGANS: A REVIEW.. Acta Cytol.

[OCR_00574] KLEIN G., REVESZ L. (1953). Quantitative studies on the multiplication of neoplastic cells in vivo. I. Growth curves of the Ehrlich and MC1M ascites tumors.. J Natl Cancer Inst.

[OCR_00578] Lala P. K., Patt H. M. (1966). Cytokinetic analysis of tumor growth.. Proc Natl Acad Sci U S A.

[OCR_00580] MENDELSOHN M. L. (1962). Autoradiographic analysis of cell proliferation in spontaneous breast cancer of C3H mouse. III. The growth fraction.. J Natl Cancer Inst.

[OCR_00582] REVESZ L., KLEIN G. (1954). Quantitative studies on the multiplication of neoplastic cells in vivo. II. Growth curves of three ascites lymphomas.. J Natl Cancer Inst.

[OCR_00585] Rosso R., Donelli M. G., Garattini S. (1967). Studies on cancer dissemination.. Cancer Res.

[OCR_00587] Smith H., Williams A. E., Lowery R. S., Keppie J. (1968). The derivation of sublines of low and high malignancy from rat and mouse tumours.. Br J Cancer.

[OCR_00592] Sylvén B., Holmberg B. (1965). On the structure and biological effects of a newly-discovered cytotoxic polypeptide in tumor fluid.. Eur J Cancer.

[OCR_00598] WHEATLEY D. N., AMBROSE E. J. (1964). TUMOUR CELL INVASION FROM TRANSPLANTABLE ASCITES TUMOURS INTO HOST TISSUES.. Br J Cancer.

[OCR_00599] WHEATLEY D. N., EASTY G. C. (1964). THE GROWTH AND INFILTRATION OF EHRLICH'S ASCITES TUMOUR IN MICE WITH REDUCED IMMUNOLOGICAL RESPONSES.. Br J Cancer.

